# Contribution of the Low-Density Lipoprotein Receptor Family to Breast Cancer Progression

**DOI:** 10.3389/fonc.2020.00882

**Published:** 2020-07-30

**Authors:** Océane Campion, Tesnim Al Khalifa, Benoit Langlois, Jessica Thevenard-Devy, Stéphanie Salesse, Katia Savary, Christophe Schneider, Nicolas Etique, Stéphane Dedieu, Jérôme Devy

**Affiliations:** ^1^Université de Reims Champagne-Ardenne (URCA), Reims, France; ^2^CNRS UMR 7369, Matrice Extracellulaire et Dynamique Cellulaire, MEDyC, Reims, France

**Keywords:** LDLR, breast cancer, microenvironment, biomarker, therapeutic targets

## Abstract

The low-density lipoprotein receptor (LDLR) family comprises 14 single-transmembrane receptors sharing structural homology and common repeats. These receptors specifically recognize and internalize various extracellular ligands either alone or complexed with membrane-spanning co-receptors that are then sorted for lysosomal degradation or cell-surface recovery. As multifunctional endocytic receptors, some LDLR members from the core family were first considered as potential tumor suppressors due to their clearance activity against extracellular matrix-degrading enzymes. LDLRs are also involved in pleiotropic functions including growth factor signaling, matricellular proteins, and cell matrix adhesion turnover and chemoattraction, thereby affecting both tumor cells and their surrounding microenvironment. Therefore, their roles could appear controversial and dependent on the malignancy state. In this review, recent advances highlighting the contribution of LDLR members to breast cancer progression are discussed with focus on (1) specific expression patterns of these receptors in primary cancers or distant metastasis and (2) emerging mechanisms and signaling pathways. In addition, potential diagnosis and therapeutic options are proposed.

## The Low-Density Lipoprotein Receptor Family and Breast Cancer: a State of Art

The low-density lipoprotein receptor (LDLR) gene family encodes single-spanning transmembrane receptors usually referred to as LDLR-related proteins (LRPs). The 14 described members are LDLR, VLDLR, LRP1/CD91/A2MR, LRP1B, LRP2/megalin/GP330, LRP3, LRP4/MEGF7, LRP5, LRP6, LRP8/ApoER2, LRP10/LRP9, LRP11/SorLA LRP12/ST7, and LRAD3 (see Table 1). Despite various homology levels, most members are clustered type I receptors sharing structural motifs (e.g., cysteine-rich complement-type repeats), involved in specific recognition of extracellular ligands, EGF-precursor homologous and β-propeller domains critical for protein folding, and pH-dependent lysosomal release of ligands. The short intracellular tail encompasses motifs allowing the recruitment of scaffolds driving the endocytic machinery and intracellular signaling. The LDLR founding member was first identified as a frequently mutated etiological factor of familial hypercholesterolemia. LDLR functions were then extended to numerous physiopathological contexts such as vascular integrity, neurobiology, and cancer development due to their peculiar ability to control membrane compartmentalization of receptors and clearance of various classes of extracellular ligands. Some LRPs were thus implicated in the specific recognition of above 50 extracellular factors, among which several growth- or migration-regulatory molecules located in the tumor microenvironment (TME) of various cancers, including mammary cancers.

**Table 1:** The 14 members of the low-density lipoprotein receptor family and their involvement in breast cancer.

**LRP isotype**	**Alternative name**	**MW (kDa)**	**Tissue distribution**	**How involved in breast cancer**
LDLR		120-160	Ubiquitous	• Overexpressed in HER2^+^ and TNBC (MDA-MB-231) (1) • Overexpression accelerates LDL cholesterol-mediated BC growth in mouse models of hyperlipidemia (2)
VLDL-R *(type II)*		96	Abundant in heart, skeletal muscle, ovary and kidney	• Up-regulated expression correlates with BC metastasis (3) • Promotes BC cell migration by up-regulating VEGF, MMP2 and MMP7 (4) • Survival factor in TNBC (5, 6)
LRAD3	Ldlrad3	50	Neurons	None
LRP1	α2MR APOER CD91	600	Ubiquitous (liver, brain, adipose tissue, fibroblasts and tissue stroma)	• Overexpressed in aggressive HER2^+^ and TNBC and associated with increased invasion (7) • Stimulates TNBC migration (8) • C766T polymorphism is suspected to increase risk of BC development (9)
LRP1B	LRP-DIT	515	Especially in brain, thyroid, skeletal muscles, testis, ovary, colon	• Considered as tumor suppressor in several cancer types but not in BC • Intracellular nuclear localization correlates with poor prognosis in invasive ductal BC (10)
LRP2	Megalin GP330	517/600	Placenta, kidney, mammary epithelial cells	• High mRNA levels in invasive BC (11) • Mutated in circulating tumor cells from BC (12) • Upregulated in T-47D (13)
LRP3	hLRP105	105	Widely expressed (ovary, heart, brain, liver, pancreas, prostate and small intestine, skeletal muscle)	ND
LRP4	MEGF7	212	Bone, cartilage, muscle, brain	ND
LRP5	LR3 LRP7	216	Widely expressed (including in mammary epithelium) with high expression in liver	• Overexpressed in TNBC and basal-like BC (14, 15) • Stimulates STK40 expression and cell viability in TNBC (15) • Regulates glucose uptake in mammary epithelial cells (16)
LRP6		180	Co-expressed with LRP5 during embryogenesis and in adult tissues	• Overexpressed in TNBC and basal-like BC (14) • Role in TNBC cell migration and invasion (MDA-MB-231 and BT549) (15) • Increases the pool of stem cells in TNBC (17)
LRP8	APOER2	106	Brain, placenta, platelets	• Overexpressed in TNBC and ER^−^ BC (18, 19) • Positive regulator of BC stem cells in TNBC, supports chemoresistance and metastasis (18) • Suggested as novel therapeutic target in TNBC (6)
LRP10	LRP9 in mouse	76	Ubiquitous (Leukocyte, lung, placenta, small intestine, liver, kidney, spleen, thymus, colon, skeletal muscle, heart)	ND
LRP11	sorLA LR11	53	Substantial levels in kidney, testis, ovary, lymph nodes, vascular smooth muscle cells and nervous system	• Drives resistance to anti-HER2 therapy (20)
LRP12	ST7	94	Heart, skeletal muscle, brain, lung, placenta and pancreas	ND

*The family core members are underlined in green, the structurally distant members are in blue, and the most distant members are in orange. ND: not determined*.

Breast cancer (BC) is one of the most diagnosed cancers among women worldwide and is the second-leading cause of cancer death. Based on their histological features, breast tumors are divided into two subtypes, *in situ* breast carcinoma and invasive breast carcinoma. The first subtype is sub-classified as either ductal (DCIS) or lobular carcinoma *in situ* (LCIS). Invasive carcinomas are further categorized into several histological subtypes, such as infiltrating ductal, invasive lobular, ductal/lobular, mucinous (colloid), tubular, medullary, and papillary carcinomas. Classification of BC based on molecular components is more useful for treatment planning and development of targeted therapies. In this classification, BC is mainly divided into hormone-receptor positive (ER^+^/PR^+^), human epidermal growth factor receptor-2 overexpressing (HER2^+^), and triple negative (TNBC). Over the past decades, breakthroughs have been made in BC treatment including surgery, radiotherapy, chemotherapy, endocrine therapy, targeted therapy, and immunotherapy. Despite all these therapeutic options, TNBC remains associated with poor outcomes and a historical lack of targeted therapies. Regarding metastases from BC, the most common first site of distant spread is bone (41%), followed by lung (22%), brain (7%), and liver (7%). The remaining 20% of patients have multiple metastatic sites (21). In this review, our focus will be on the role played by the members of the LDLR family in BC by examining specifically their implications within the tumor microenvironment. The clinical relevance of targeting these receptors for developing new targeted therapies will also be discussed.

## LRPS and Breast Cancer Cells: a Close and Complex Relationship

Obesity and hypercholesterolemia are risk factors for BC that negatively impact therapeutic efficacy (22, 23). Higher levels of plasmatic cholesterol, LDL (low-density lipoprotein), and triglycerides and low circulating levels of HDL are frequently found in patients with BC (24). Interestingly, LDL was reported to affect the sensitivity of tumor cells to radiotherapy in inflammatory BC (25). LDL could affect the adhesion of BC cells involved in cell migration and proliferation and a difference in the quantity and type of lipid synthesis and storage has been demonstrated in basal-like ER^−^ compared to luminal ER^+^ BC cells (26). Patients with BC usually exhibit elevated serum levels of oxidized LDL, reported to induce structural DNA alterations, a decrease in DNA repair, and pro-oncogenic signaling pathways (1).

In mammary tumor tissues, LDLR expression is higher and cholesteryl ester accumulation is associated with an increase of Ki67 expression and poor clinical outcome (27, 28). BC cells express higher LRP1 and LDLR, allowing a better uptake of LDL-C from the blood (29). Cholesterol may also generate 27-hydroxycholesterol, an estrogen mimetic involved in epithelial-to-mesenchymal transition (EMT) in ER^+^ BC cells (30, 31). In addition, LDLR and acyl-CoA:cholesterol acyltransferase-1 are increased in HER2-positive and triple-negative tumors compared to luminal A tumors (1).

Among LDLR, the giant receptors are represented by LRP1, LRP1B, and LRP2, sharing strong structure homologies but showing discrepancies in terms of endocytic kinetics and expression pattern (32). LRP2/Megalin is required for the internalization of vitamin D and its activation to 1,25-OH vitamin D. A reduced expression was found in some BC, leading to a decrease of its nuclear receptor VDR activation, which plays an important anti-proliferative role (33). *LRP2* mRNA was also detected at fairly high levels in invasive BC but with extremely high variability (11).

LRP1B, a close homolog of LRP1, is among the top 10 significantly mutated genes in human cancer (34, 35). LRP1B is mutated in circulating tumor cells from BC and may participate in human mammary gland carcinogenesis (12). The nuclear localization of its intracellular domain is significantly related to poor prognosis in patients with invasive ductal breast carcinoma and to a significant decrease of both disease-free and overall survival in patients with luminal A type breast carcinoma (10).

LRP1 was initially identified as a tumor suppressor controlling, by endocytic clearance, the extracellular matrix-degrading enzymes in the microenvironment of various invasive tumors (36). In BC models, α2-macroglobulin/LRP1-dependent uptake of pepsin is involved in the control of the invasive potential of luminal and TNBC cells (37). However, other studies support a more complex view of LRP1 functions in tumor cells. The serine protease inhibitor PN-1/SerpinE2, which is highly expressed in ER^−^ and high-grade BC, stimulates lung metastasis of mammary tumor cells through LRP1-dependent secretion of MMP-9 (38). By contrast, SerpinE2 and LRP1 were identified among the genes induced by ZEB-1, an EMT driver that limits the expression of LRP1-targeting miRNAs, thereby triggering tumor cell autocrine factors that predict poor survival in early stage of BC (39). LRP1 can exert a dramatic control of tumor cell plasticity and migratory capacities. Its silencing in TNBC cells increased cellular rigidity, decreased cellular protrusions, and finally impaired migration (8). Converging data illustrate the important role of Hsp90α binding to LRP1 during EMT-related events in BC (40–43). Hypoxia leads to HIF1-α-dependent secretion of Hsp90α by TNBC cells. Its specific binding to LRP1 stimulates tumor development and metastatic lung colonization (42). This interaction and subsequent pro-metastatic signals are reinforced by clusterin in both luminal and TNBC models (43). Interestingly, within extracellular space, Hsp90α is absent from the normal microenvironment, suggesting promising opportunities for targeted therapy (42). These studies underline the versatility of LRP1 functions in breast TME and support ongoing research to identify the specific molecular interfaces mobilized by the receptor that could be targeted to control aggressive behavior of tumor cells. A less characterized member of LRPs, SorLA/LRP11, was recently involved in the endocytic trafficking of HER2 (20). The depletion of SorLA was reported to affect lysosomal function and sensitize HER2-overexpressing cells resistant to targeted therapy. Its targeting could therefore affect compartmentalized pools of oncogenic receptors and restore efficacy of conventional treatments.

LRP5 and LRP6, as co-receptors of the Wnt/β-catenin pathway, are directly involved in breast tumorigenesis. Wnt ligands such as frizzled homolog 7 and LRP6 are overexpressed in TNBC (44), whereas Wnt antagonists are frequently silenced by methylation in BC (45). Blockade or silencing of LRP6 in SUM1315 basal BC cells results in a re-expression of epithelial markers and a decreased capacity to self-renew and metastasize (46). Similarly, LRP6 downregulation in MDA-MB-231 decreases the pool of BC stem cells (17). These effects are more pronounced on TNBC cell migration and invasion (47). The use of benzimidazole compounds on TNBC cells exerts anticancer activity by inhibiting the Wnt/β-catenin pathway. Prodigiosin and other compounds decrease the phosphorylation of LRP6 (active form), and inhibit the activation of mTORC and Wnt/βcatenin signaling (48–52). The disruption of lipid rafts in TNBC cells is associated with a decrease of LRP6 and β-catenin expression, cell proliferation, and migration (53). Besides this Wnt/βcatenin canonical pathway, LRP5 was also reported to be involved in the uptake of glucose in mammary epithelial cells, through Apolipoprotein E (ApoE) binding. The glucose uptake is essential for regulating the growth rate of these cells (16). A soluble LRP6 ectodomain can prevent tumor progression, by inhibiting cell migration and metastasis, by limiting the Frz-mediated non-canonical pathway activation in breast tumor cells (54).

**Figure 1:**
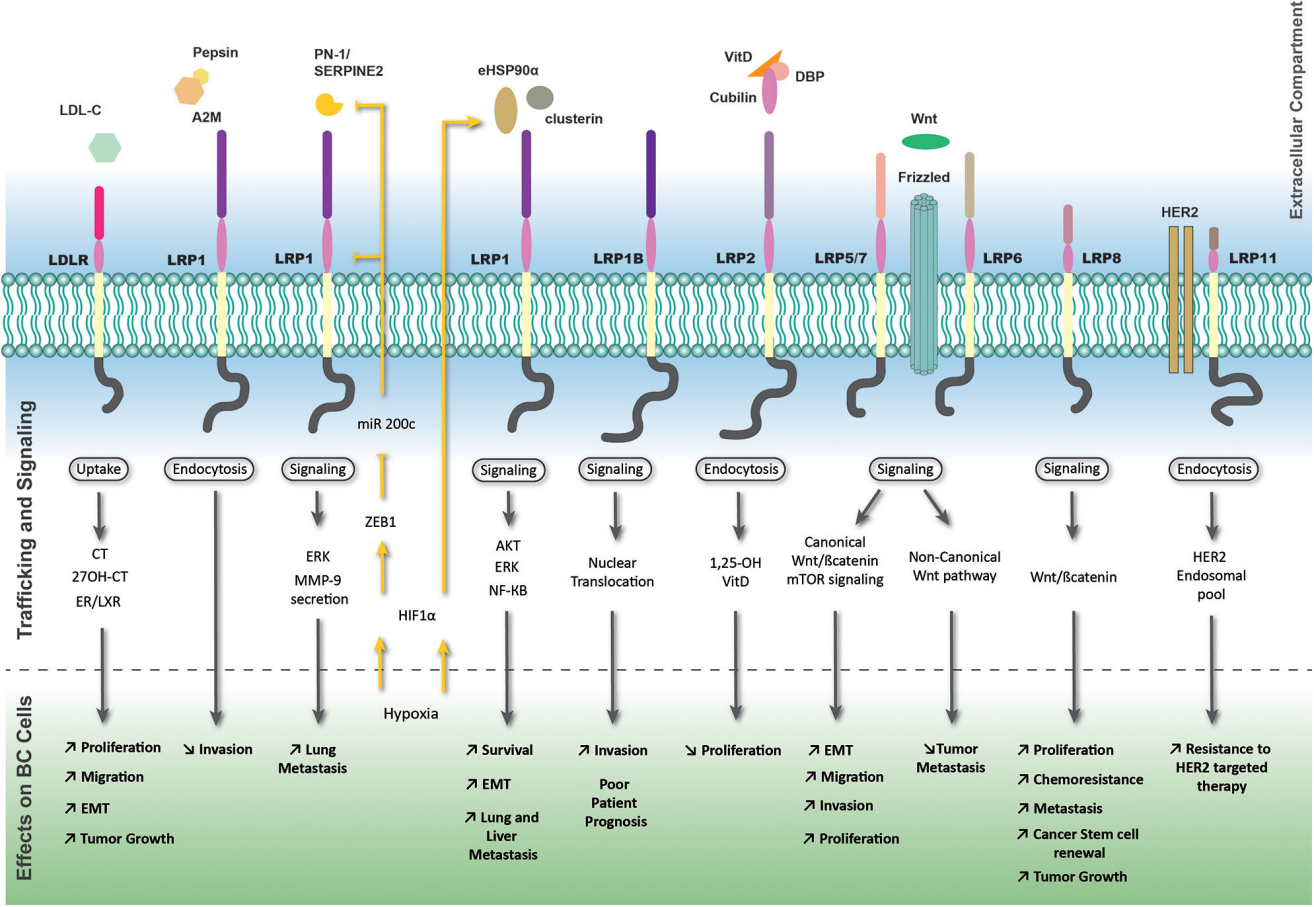
LRP-mediated signaling pathways and trafficking in breast tumor cells. The most important members of the LDLR family exhibiting effects on breast cancer cells are represented with their associated extracellular ligands. Outside-in and inside-out pathways are represented by black and yellow arrows or lines, respectively. The yellow strikethrough line indicates an inhibition. A2M, alpha-2 macroglobulin; CT, cholesterol; ER, estrogen receptor; LXR, liver X receptor; VitD, vitamin D.

The function of LRP8/ApoER2, strongly expressed in ER negative breast tumors was recently described in breast tumor initiating cells, which constitute a clinical challenge of the pathology (18). Interestingly, its depletion impairs TNBC cell proliferation and promotes apoptosis (19). LRP8 depletion also leads to Wnt/β-catenin signaling inhibition, decreases the pool of BC cells, limits their tumorigenic potential in murine xenografts, and finally restores TNBC cell sensitivity to chemotherapy (18). An overview of the complex and multiple LRPs-mediated signaling pathways is shown in Figure 1.

## Functional Interplay Between LRPS and Cells Within the Tumor Microenvironment

The breast TME encompasses multiple cell types including fibroblasts, immune cells, adipocytes, and endothelial cells (55). In human breast tumors, fibroblasts are the most abundant stromal cells and high levels of LRP1 expression was reported (56). In fibroblasts, LRP1 binds to CTGF, PDGF, and TGFβ and interacts with their respective receptors, thereby modulating their mitogenic or contractile capacities (57–61). Similarly, to LRP1, LRP6 interacts closely with PDGFRβ and TGF-βRI in pericytes and is involved in their trans-differentiation into myofibroblasts in response to TGFβ or CTGF. Therefore, it stimulates the PDGF-BB-dependent proliferation of established myofibroblasts via β-catenin-independent mechanisms (62). Likewise, Wnt7a secreted by aggressive breast tumor cells promotes the activation of stromal fibroblasts through TGFβ signaling (63). In cancer-associated fibroblast from mammary tumors, the stabilization of LRP6 at cell surface by DKK3 stimulates β-catenin and YAP/TAZ signals, promoting pro-tumorigenic functions such as ECM stiffening (64). Interestingly, pro-cath-D hypersecreted by cancer cells in the breast TME stimulates fibroblast outgrowth by inhibiting the release of LRP1β (intracellular domain), which is able to regulate gene transcription (65).

Adipocytes are mainly engaged during BC progression through a metabolic crosstalk with adjacent tumor cells and adopt a modified phenotype called cancer-associated adipocytes (66). Resulting dysfunctional adipocytes overexpressed fatty acid, cholesterol, triglycerides, hormones, but also adipokines, inflammatory cytokines, and proteases that are linked to cancer progression (66). LRP-1 is highly expressed in preadipocytes and is involved in adipocyte differentiation, especially through its regulation of peroxisome proliferator-activated receptor γ (67). LRP1 has also been demonstrated to be upregulated in obese mouse adipocytes and obese human adipose tissues (67) and to regulate insulin receptor and GLUT4 trafficking and activation (68, 69). Through modulation of Wnt5a signaling, LRP1 controls cholesterol storage and fatty acid synthesis during adipocyte differentiation (70). An attenuated endocytosis of apoA5 by adipocytes was demonstrated in both adipose tissue from obese patients and insulin-resistant adipocytes. The mechanism underlying this phenomenon might be related to a reduced endocytic activity of LRP1 and/or an attenuated insulin-dependent movement of LRP1 from intracellular structures to the cell surface (71). These mechanisms may lead to excessive augmentation of triglyceride storage and abnormal metabolism of adipocytes, hence promoting the development of obesity and obesity-associated disorders such as BC.

LRP1 is also abundantly expressed by various immune cells and its function in HSP-mediated antigen presentation and subsequent innate immune response is well described in macrophages and dendritic cells (72). LRP1 also inhibits macrophage-driven inflammation by decreasing cell-surface abundance of the TNF receptor-1 and Iκ-B kinase/NF-κB intracellular activation (73). By contrast, production of sLRP1 (shed or soluble LRP1) by macrophages induces pro-inflammatory factor synthesis such as IL-10, TNF-α, and MCP-1 (74). Macrophage inflammatory protein-1a/CCL3, known to amplify inflammation, is overproduced in the absence of LRP1 in myeloid cells, leading to enhanced CCR5-expressing monocyte recruitment to tumors and cancer angiogenesis (75).

Recent studies have demonstrated the crucial angio-modulatory actions of LRP family members in various solid tumors, including BCs (39, 51, 76–79). LDL and VLDL (very low density lipoprotein) are involved in the secretion of diffusible angiogenic factors by BC cells, such as amphiregulin (79). Moreover, the binding of Wnt3a to LRP6 stimulates VEGF production by TNBC cells (51). The stoichiometry of Wnt ligands and their secreted regulators such as Dickkopfs (DKK) seems instrumental to fine-tune LRP5/6 functions in the TME. DKK1 was indeed described as anti-angiogenic, whereas DKK2 binding to LRP6 triggers potent induction of endothelial cell sprouting (80). LRP1 is widely expressed in various endothelial cells and its specific binding to tPA alone or complexed with uPA/PAI-1 induces vascular permeability in the blood–brain barrier (BBB) or in lung microvasculature, two major sites of BC cell metastatic homing (81, 82). LRP1 controls multiple aspects of endothelial cell metabolism (83) and participates to the control of intercellular junctionality, morphogenesis, and proliferation (81, 84, 85). Interestingly and as stated above, during epithelial-to-mesenchymal transition in breast tumors, LRP1 expression was derepressed through ZEB-1-dependent inhibition of LRP1-targeting miRNAs, thereby contributing to vascular mimicry of breast tumor cells (39). The induction of this endocytic receptor could thus reinforce endothelial interface of breast tumor cells and facilitate their metastatic dissemination.

## Clinical Significance of LRPS as Biomarker in the Context of Breast Tumors

A large-scale study conducted on solid tumors indicates that mostly LDLR mRNA are overexpressed in breast invasive carcinomas with *LRP2* mRNA being the most expressed, but no correlation with patient survival was observed (11). Only a few studies are focused on LRP1 expression in BC patient samples. LRP1 was first immunohistochemically studied in fresh frozen tissue from primary invasive breast carcinomas, ductal *in situ* carcinomas, and auxiliary lymph-node metastases in 1996 (56). LRP1 staining appeared intense in all stromal fibroblasts both outside and within the tumor tissue and scattered in macrophages and mast cells. Interestingly, epithelial cells, endothelial cells, and lymphocytes appeared negative for LRP1. A more recent immunohistochemistry study of LRP1 expression, performed on infiltrating ductal breast carcinomas, brought different results as cytoplasmic LRP1 overexpression was identified in tumor cells in addition to non-neoplastic stromal cells, whereas normal ductal cells were always negative (7). Concerning *LRP1* polymorphism, although C766T mutation was firstly reported as significantly higher in patients with BC (9), the increased risk of BC development associated to LRP1 polymorphism is not definitely established. Of note, neoadjuvant chemotherapy in BC did not impair LRP1 expression (28).

## A Better Understanding of LRP Functionalities May Lead to Efficient Therapeutic Strategies

Therapeutic approaches involving LRPs developed in oncology, particularly in BC, aim to address the endocytic properties of these receptors as vectorization tools. One of the remaining therapeutic concerns for BC patients is related to metastases. Brain metastases occur in about 15–30% of women with stage IV BC. The targeting of the BBB, formed by endothelial cells, astrocytes, and pericytes embedded in the capillary basement membrane, remains critical for treating brain metastases. As LRP1 transports ligands such as β-Amyloid or tPA across the BBB and is expressed at high levels in this tissue (86), it appears as a promising candidate for targeted therapy against metastatic BC cells. In this context, the main therapeutic approaches use the Angiopep-2, an LRP1 binding peptide first identified by Demeule and colleagues (87). Combined with three paclitaxel residues, this molecule (namely, GRN1005, formerly known as ANG1005) binds to LRP1, crosses the BBB, and allows a better drug delivery in the brain compartment (78, 87). Phase I/II clinical trials with ANG1005/GRN1005 show that treatment is safe and brings clinical benefit for both peripheral metastatic BC and brain metastasis, even if the tumor had previously developed resistance to conventional taxanes. Interestingly, an open-label Phase III study will start in 2020 to investigate whether ANG1005 can prolong patient survival in HER2-negative BC patients with newly diagnosed leptomeningeal disease and previously treated brain metastases (NCT03613181). Angiopep-2 can also be useful to target BC cells overexpressing LRP1. For instance, Angiopep-2 was used to decorate nanoparticules combined with doxycycline (Angio-DOX-DGL-GNP) in TNBC to facilitate the drug penetration and accumulation in BC cells (88).

The endocytic properties of LRP2 have also been used to improve the effectiveness of anticancer drugs in resistant BC cells (89). In this context, lipid-polyethylenimine hybrid nanocarriers decorated with apolipoprotein E (Ap-LPN) were developed for improving siRNA delivery against clusterin in MCF7 BC cells, leading to increased cell chemosensitization toward paclitaxel.

Another strategy of tumor targeting was used with the NT4 peptide, a tetrabranched peptide from the human neurotensin, capable of binding LRP1 and LRP6 by mimicking ApoE and midkine heparin binding site (90). Depau and collaborators showed that methotrexate conjugated with NT4 can overcome drug resistance in methotrexate-resistant human BC cells (91). NT4 conjugated with other drugs (NT4-paclitaxel, NT4-5FdU) were tested in various animal models of human cancer, including an orthotopic mouse model of human BC, leading to improved drug activity as compared to unconjugated counterpart (92–94).

More recently, some LRPs have been identified as direct molecular targets for BC. LRP6 is probably the most promising target in the TNBC with its overexpression leading to Wnt signaling pathway activation together with tumorigenesis promotion (5). Several drugs such as salinomycin, prodigiosin, and niclosamide indeed induce LRP6 phosphorylation and degradation leading to decreased tumor growth (49, 50, 95). Ren and collaborators have suggested that soluble LRP6 ectodomain could also be used as an innovative anti-metastatic drug (54).

## Conclusion and Subjective Points of View

Receptors from the LDLR family are increasingly emerging as key relevant biomarkers in oncology and potential therapeutic targets. Their multiple implications within the TME, variable expression related to tumor stages, together with molecular versatility, constitute the main challenges to better understand their functionalities. In breast cancer, scientific evidence is fragmented, sometimes contradictory, and only a few clinical data are available. Potential prognostic value of these receptors is still unclear, thus preventing from demonstrating clinical benefits. Additional studies will be necessary to establish a link between LRPs and some events promoting obesity or metabolic diseases, particularly to improve the treatment of BC in post-menopausal patients. LRP1 is likely to be the most promising receptor because it constitutes an efficient drug carrier within tumor cells. Very promising trials are ongoing in HER2-negative BC patients with metastasis. In addition, LRP1 could also be considered as an attractive therapeutic target in TNBC. However, its high molecular weight, intricate regulation, and sub-cellular localization together with its ability to bind multiple extracellular ligands within the same clusters, make current research extremely complex and can lead to contradictory conclusions. The use of more advanced *in vitro* multi-cellular and 3D tumor-based systems (tumoroïds) with patient-derived cells will be key to deeper understand the functionality of this receptor. In the coming years and in order to consider LRP1 as an innovative vectorization tool, the approach should be focused on the endocytic properties of overexpressed LRP1 rather than on the modulation (e.g., inhibition or reduction) of LRP1 expression.

## Author Contributions

All authors listed have made a substantial, direct and intellectual contribution to the work, and approved it for publication.

## Conflict of Interest

The authors declare that the research was conducted in the absence of any commercial or financial relationships that could be construed as a potential conflict of interest.

## Abbreviations

BBB: blood–brain barrier
BC: breast cancer
DCIS: Ductal carcinoma *in situ*
ECM: extracellular matrix
EMT: epithelial-to-mesenchymal transition
ER: estrogen receptor
HDL: high-density lipoprotein
HER2: human epidermal growth factor receptor-2
LCIS: lobular carcinoma *in situ*
LDL: low-density lipoprotein
LDLR: low-density lipoprotein receptor
LRP: LDL receptor-related protein
PR: progesterone receptor
TME: tumor microenvironment
TNBC: triple-negative breast cancer
VDR: vitamin D receptor
VLDL: very low density lipoprotein.
